# Transcriptome‐Based Classification of Resected Pancreatic Ductal Adenocarcinoma Enhances Prognostic Modelling Accuracy of Overall Survival Following Adjuvant Treatment

**DOI:** 10.1002/ijc.70519

**Published:** 2026-04-27

**Authors:** Marjolein F. Lansbergen, Vincent R. Lanting, Paul Manoukian, Marc G. Besselink, Geert Kazemier, Ignace H. J. T. de Hingh, Mike S. L. Liem, Casper H. J. van Eijck, Erwin van der Harst, Vincent E. de Meijer, Ronald M. van Dam, Martijn W. J. Stommel, Jan Koster, Michael W. T. Tanck, Arantza Fariña Sarasqueta, Joanne Verheij, Frederike Dijk, Johanna W. Wilmink, Maarten F. Bijlsma, Hanneke W. M. van Laarhoven, Dutch Pancreatic Cancer Group

**Affiliations:** ^1^ Department of Medical Oncology, Amsterdam UMC University of Amsterdam Amsterdam the Netherlands; ^2^ Laboratory of Experimental Oncology and Radiobiology, Amsterdam UMC University of Amsterdam Amsterdam the Netherlands; ^3^ Cancer Center Amsterdam, Imaging and Biomarkers Amsterdam the Netherlands; ^4^ Department of Vascular Medicine, Amsterdam UMC University of Amsterdam Amsterdam the Netherlands; ^5^ Amsterdam Cardiovascular Sciences Amsterdam the Netherlands; ^6^ Department of Surgery, Amsterdam UMC University of Amsterdam Amsterdam the Netherlands; ^7^ Department of Surgery, Amsterdam UMC Vrije Universiteit Amsterdam the Netherlands; ^8^ Department of Surgery Catharina Hospital Eindhoven the Netherlands; ^9^ Department of Epidemiology, GROW‐School for Oncology and Developmental Biology Maastricht University Maastricht the Netherlands; ^10^ Department of Surgery Medisch Spectrum Twente Enschede the Netherlands; ^11^ Department of Surgery Erasmus Medical Center Rotterdam the Netherlands; ^12^ Department of Surgery Maasstad Hospital Rotterdam the Netherlands; ^13^ Department of Surgery University Medical Center Groningen Groningen the Netherlands; ^14^ Department of Surgery Maastricht University Medical Center Maastricht the Netherlands; ^15^ Department of Surgery Radboud University Medical Center Nijmegen the Netherlands; ^16^ Department of Epidemiology and Data Science, Amsterdam UMC University of Amsterdam Amsterdam the Netherlands; ^17^ Department of Pathology, Amsterdam UMC University of Amsterdam Amsterdam the Netherlands

**Keywords:** adjuvant chemotherapy, pancreatic cancer, resection, RNA sequencing, subtypes

## Abstract

In pancreatic ductal adenocarcinoma, patient outcomes after resection remain highly variable. Prognostic models are often inaccurate. Our study aimed to improve survival prediction by adding transcriptome‐based classification to a validated prognostic model and applying it on a multicenter real‐world cohort of fresh‐frozen resection materials. RNA was sequenced if tumor cellularity was > 30%. The samples were classified using transcriptome‐based classification. Survival differences between transcriptome‐based subtypes were studied in patients treated with and without adjuvant chemotherapy. 25.6% of the patients received neoadjuvant treatment (NAT). Samples of 461 patients were collected, of which 118 samples underwent RNA sequencing. Of those, 39.0% had a basal‐like subtype and 61.0% had a classical subtype. The basal‐like subtype became dominant after NAT (63.3%, *p* = 0.004). Patients with a classical tumor survived longer than those with a basal‐like tumor (median overall survival [OS]: 22.8 vs. 11.4 months; *p* < 0.001, in patients receiving adjuvant gemcitabine, and 10.7 vs. 5.4 months; *p* = 0.082, in patients without adjuvant treatment). In multivariable Cox regression, the classical subtype significantly associated with increased survival (hazard ratio = 0.38; *p* = 0.002) and adding transcriptome‐based subtyping significantly improved the prognostic model (*p* = 0.002). Subtype and adjuvant treatment independently significantly associated with OS. Transcriptome‐based subtyping significantly adds to clinical variables in survival prediction after surgery. The independent associations for subtype and adjuvant treatment with OS indicate that subtypes are prognostic, but not predictive for OS with adjuvant treatment. The provided prognostic information could potentially support treatment decisions and serve as stratification factor.

## Abstract (highlights)

What's new?

Outcomes after resection of pancreatic ductal adenocarcinoma remain highly variable, and existing prognostic models lack sufficient accuracy. Here, the authors aimed to improve survival prediction by integrating transcriptome‐based subtyping into a validated clinical model and applying it to a multicenter real‐world cohort of fresh‐frozen resection materials. The results demonstrate that incorporating molecular subtyping significantly improves prognostic performance, including in patients receiving adjuvant chemotherapy. This study highlights the potential of transcriptomic profiling to uncover molecular heterogeneity undetected by traditional pathology as well as to support treatment decisions and serve as a stratification factor in clinical trials.

## Graphical abstract

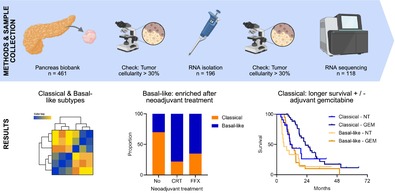

**Abbreviations**
AIC: Akaike information criterion
ANOVA: analysis of variance
ASA: American Society of Anesthesiology
BIC: Bayesian information criterion
BMI: body mass index
CI: confidence interval
CK5: keratin‐5
CRT: chemotherapy (gemcitabine) combined with radiation
df: degrees of freedom
DFS: disease‐free survival
DNA: deoxyribonucleic acid
ECOG: Eastern Cooperative Oncology Group
ESTIMATE: Estimation of STromal and Immune cells in MAlignant Tumor tissues using Expression data
EUS: endoscopic ultrasound
FOLFIRINOX: 5‐fluorouracil, folinic acid, irinotecan, oxaliplatin
GATA6: GATA‐binding factor 6
HE: hematoxylin eosin
HMF: Hartwig Medical Foundation
HR: hazard ratio
IHC: immunohistochemistry
LNR: lymph node ratio
miRNA: micro ribonucleic acid
NA: not available
NAT: neoadjuvant treatment
OS: overall survival
PCA: principal component analysis
PDAC: pancreatic ductal adenocarcinoma
PurIST: Purity‐Independent Subtyping of Tumors
RIN: RNA integrity
RNA: ribonucleic acid
RNA‐Seq: RNA sequencing
R‐status: resection margin status
UICC: union for international cancer control

## 1 Introduction

The range of interventions for patients with resectable and borderline resectable pancreatic ductal adenocarcinoma (PDAC) has expanded in the past decade to include both neoadjuvant (NAT) and adjuvant chemotherapy. Multiple efforts have been undertaken in the preoperative setting to increase resectability and survival rates [1, 2, 3, 4]. In the adjuvant setting, the introduction of gemcitabine combined with capecitabine and the combination of 5‐fluoruoracil, leucovorin, irinotecan, and oxaliplatin (FOLFIRINOX) caused increased survival rates as well [5, 6]. Despite these improvements, patients with resected PDAC still have a poor prognosis. Notably, overall survival variability is substantial, as evidenced by interquartile ranges of more than 40 months for overall survival (OS) [6]. Given this heterogeneity, individualized prognostication could provide patients with information and benefit patient stratification in clinical trials.

Several prognostic models for outcome after pancreatic resection exist, which include clinical or pathological variables, such as performance status, differentiation grade and lymph node status. However, most prognostic models showed modest discriminatory ability to distinguish between good and poor responders, often showing inconsistent performance between derivation and validation cohorts [7]. In addition to pathological variables, transcriptome‐based subtyping might contribute to survival prediction after surgery. Several transcriptome‐based classification systems have been developed on resection material. These classifiers generally identify a less aggressive epithelial or classical subtype, and a more aggressive squamous, mesenchymal or basal‐like subtype [8, 9, 10, 11]. To adapt these cluster‐based classifiers for clinical application, the PurIST classifier has been developed as a single‐sample classifier suitable for multiple types of material [12].

When this classifier is applied in univariable analyses on historical cohorts, prognosis‐related subgroups are consistently identified. However, studies on the prognostic value of this classifier lack samples that were exposed to systemic therapy before or after surgery or did not include multivariable analyses incorporating other prognostic variables [12, 13]. Previously it was suggested that pretreatment with chemotherapy would induce basal‐like gene expression [14] or “neural‐like progenitor” gene expression [15], which might affect subtyping. The aim of this study was to assess the benefit of transcriptome‐based classification, when added to pathological variables, in modeling of survival after surgery (with or without NAT) and adjuvant chemotherapy and thereby improving patient counseling and stratification in the future.

## 2 Methods

### 2.1 Study Population and Clinical Data Collection

Fresh frozen tumor tissue from resection specimens was collected from patients with pancreatic ductal carcinoma, including its histomorphological subtypes, from 2015 to 2022. All available samples with known diagnosis were collected. Material was obtained from local biobanks collaborating with the Dutch Pancreas Parel Biobank, incorporated in the Pearl String Initiative [16], and from the Amsterdam University Medical Center local biobanks. Seven academic and three large non‐academic hospitals participated in the material collection. Clinical data was collected using Castor Electronic Data Capture in a predesigned electronic case report form [17]. T‐stage was registered according to guidelines of the 8th edition of the Union for International Cancer Control (UICC). The maximal diameter was used to convert the T‐stage from the 7th to the 8th edition if the resection took place in 2017 or earlier. R‐status was obtained from the pathology report. The tumor was considered R0 when the tumor‐free resection margin was > 1 mm and R1 when the margin was ≤ 1 mm [18]. When clinical data were missing, samples were excluded from the analysis. For feasibility reasons, clinical data were only collected for the samples that were included in RNA sequencing. The clinical data were summarized using “table1” and “stats” R packages.

### 2.2 Tissue Processing

To evaluate tumor cell percentage of the samples, hematoxylin–eosin (HE)—slides were made of all samples (*n* = 461) and assessed by a pathologist. All samples with a tumor cell percentage of 30% or higher were included. When possible, macrodissection was performed to manually select the parts of the sample with enough tumor cells. Subsequently, around 40 slides of 10 μm were cut with a 5 μm HE‐slide in the middle. Tumor RNA and DNA were isolated from these slides according to the protocol of Qiagen AllPrep DNA/RNA/miRNA kit 50 (80224). RNA concentrations were determined by NanoDrop (Thermo Scientific), TapeStation (Agilent) and Qubit assays (Thermofisher). RNA samples were submitted to Hartwig Medical Foundation (HMF) in two batches with a comparable distribution of RNA integrity number (RIN) values (3.1 in batch 1 and 3.3 in batch 2) and RNA concentrations (77 ng/μL in batch 1 and 94 ng/μL in batch 2).

### 2.3 RNA Sequencing

RNA sequencing was performed by HMF. A total of 50–100 ng of RNA was used as input for KAPA RNA HyperPrep Kit with RiboErase (Human/Mouse/Rat) library preparation (Roche), which was performed on an automated liquid handling platform (Beckman Coulter). RNA was fragmented using high temperature in the presence of magnesium to a targeted fragment length of 300 bp. Barcoded libraries were sequenced as pools on NextSeq 500 (V2.5 reagents) generating 2 × 75 read pairs or at a later stage on a NovaSeq 6000 generating 2 × 150 read pairs using default settings (Illumina). Binary base call output from the sequencing platform was converted to fastq files using bclconvert (default parameters with strict‐mode set on true). The sequencing coverage and quality statistics for each sample are summarized in Table S7. Genome build used was NCBI 37/HG19.

### 2.4 Bioinformatics

Fastq files were processed to transcript per million (tpm) by HMF using their isofox algorithm/pipeline (fully described here [19]) and were loaded into the R2 Genomics Analysis and Visualization Platform [20] for analysis. Principal component analysis (PCA) was performed as a function in R2. Because of the spread in RIN value and its effect on RNA expression values as shown by PCA plot (Figure S1B), the gene expression levels (isofox adjusted transcript per million values) were corrected for RIN value using the “removeBatchEffect” function from the limma package in R, where the RIN values were used as a continuous covariate. Next, to control the results, the RIN‐corrected gene expression profiles were clustered according to Euclidean distance based on a gene set consisting of Y‐linked genes [21] and the clustering results were compared to the sex of the patient as was assigned by the treating physician. No further batch covariates were corrected for, since visual inspection on PCA after RIN value did not exhibit appreciable differences for the different HMF batches. Samples were classified according to Purity Independent Subtyping of Tumors (PurIST) [12] and Moffitt [9] classification, using R packages “ConsensusClusterPlus” for Moffitt classification and “switchBox” for PurIST classification. Heatmaps were created by R package “ComplexHeatmap” or the heatmap function in the R2 platform. Estimation of STromal and Immune cells in MAlignant Tumor tissues using Expression data (ESTIMATE) scores for stromal and immune components were determined by the “tidyestimate” R package.

### 2.5 Effect of Neoadjuvant Treatment on Gene Signature

Relation between any type of neoadjuvant treatment and PurIST subtype was assessed using Fisher's exact test (“stats” R package). To study the effect of specific treatment regimens in more detail, patients who underwent radiotherapy with FOLFIRINOX (*n* = 1), radiotherapy without any systemic treatment (*n* = 1), an immunomodulatory agent with FOLFIRINOX (*n* = 1), or had a history of treatments against other cancer types (*n* = 4), were excluded from the analysis because of their low prevalence. Tumor cell‐intrinsic gene signatures were derived from Hwang et al. [15]. For each signature, consisting of the top 200 most differentially expressed genes, a *z*‐score was calculated in the R2 platform. *Z*‐scores were tested between groups using Kruskal‐Wallis and post hoc Dunn's testing in Graphpad Prism.

Gene signature correlation analyses were performed within the R2 platform. First, gene signatures were established for the groups of samples treated with neoadjuvant gemcitabine + radiotherapy versus no neoadjuvant treatment, and for the samples treated with neoadjuvant FOLFIRINOX versus no neoadjuvant treatment. To find differently expressed genes, analysis of variance (ANOVA) was applied using post hoc false discovery rate testing (only in the comparison neoadjuvant gemcitabine + radiotherapy vs. no neoadjuvant treatment), setting the *p* < 0.01. To normalize gene expression and compare the expression of both literature‐derived and neoadjuvant‐related signatures between samples, gene expression values were transformed into *z*‐scores. These *z*‐scores were correlated and *R*‐, *t*‐, and *p*‐values were provided. Literature‐derived gene signatures were retrieved from Hwang et al., Dijk et al., Moffitt et al., Elyada et al., Rashid et al., and the Broad Institute's Hallmark [22] and Cell type C8 2025 [23] update gene signatures.

### 2.6 Statistical Analysis

Primary outcome was overall survival (OS) in months after resection until death. The secondary outcome was disease‐free survival (DFS) in months after resection until the date of local recurrence or distant metastasis detection on imaging or the date of death. Patients who died or were lost to follow‐up within 90 days were excluded from all survival analyses to exclude patients who died because of surgical complications. Patients were censored on the last date of contact when no events were registered. The main analysis of the effect of gene expression‐based subgroup on overall survival was performed by a Cox regression model, correcting for lymph node ratio (LNR), differentiation grade, R‐status and administration of adjuvant treatment, covariates derived from the internationally validated Amsterdam model for survival prediction in pancreatic cancer after pancreatoduodenectomy [24]. The effect sizes of this model were presented as an adjusted hazard ratio with a 95% confidence interval (CI) and the analysis was performed by the “coxph” function from the “survival” package in R. For LNR, 0.2 was used as a cut‐off [25]. The proportional hazard assumption was tested for an independent correlation of Schoenfeld residuals with time (“cox.zph” function, “survival” package). This assumption was not met for the administration of adjuvant therapy, and consequently it was coded as a time‐varying variable (“survSplit” function, “survival” package). The time cut‐offs for the time groups were based on where the 95% CI of the beta coefficients included zero (first cut‐off) and where the coefficient line flattened (second cut‐off). The Cox regression models were complete‐case analyses and samples with missing variables were excluded. However, as differentiation grade was missing in a substantial part of the patients (30/118, 25.4%), mainly those receiving NAT (18/30, 60.0%), and these patients were consequently missing in the survival models, analyses were repeated with differentiation grade missing as an extra category of differentiation grade. A likelihood ratio test and the Akaike information criterion (AIC) and Bayesian information criterion (BIC) were used to compare the prognostic models with and without the transcriptome‐based subtype included (“AIC,” “BIC,” and “anova” function from “stats” package). The analysis was repeated in a selection of patients who did not receive neo‐adjuvant therapy before resection (the NAT‐naïve patients). In addition, the overall survival was compared between the transcriptome‐based subtypes using a log‐rank test for time‐to‐event outcomes and by showing a Kaplan–Meier curve (Graphpad and “survfit” function from “survival” package). Proportions were tested with either a Fisher's exact or a Chi‐squared test (“stats” package). Differences between groups in continuous variables were tested with a Mann–Whitney *U* test (“stats” package).

### 2.7 Software

Transcriptome‐based subtyping and clinical variable testing were performed in R Core Team (2020), version 4.3.2. R: A language and environment for statistical computing. R Foundation for Statistical Computing, Vienna, Austria. URL https://www.R‐project.org/. The used packages are specified in Section 2. Graphs were made in Graphpad Prism 10. Gene expression analyses were performed in the R2 Genomics Analysis and Visualization Platform [20].

## 3 Results

### 3.1 Patient Inclusion and Tissue Collection

**FIGURE 1:**
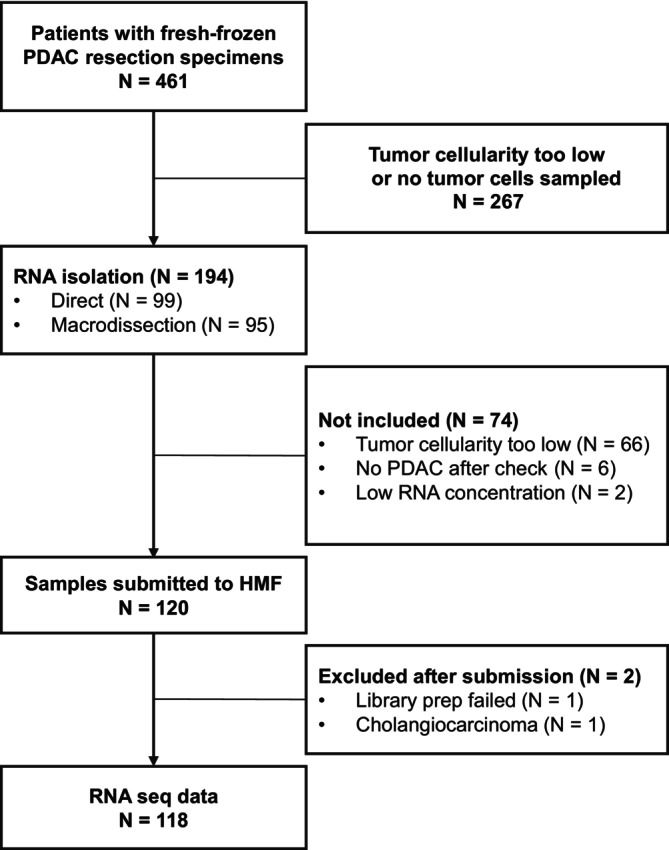
Inclusion flow chart. Fresh frozen resection specimens were obtained from institutional biobanks. Tumor cellularity was assessed twice: First, to select samples for RNA isolation and subsequently to select the RNA samples with sufficient tumor cellularity (30%) for RNA‐Seq. When collecting clinical data of the RNA‐isolated samples, some samples appeared to have another diagnosis than PDAC (“no PDAC after check”).

Tissue specimens of 461 patients were collected from 10 Dutch hospitals. Tumor samples with a sufficient tumor cell percentage, or with an opportunity to manually remove low‐tumor areas, were selected for RNA isolation. Of these 194 samples, RNA isolation could be performed directly in 99 samples, and manual macrodissection was required to increase tumor cellularity in 95 samples. After isolation and pathologic assessment of mid‐sample HE‐slides, 118 samples were submitted for RNA sequencing, presenting 25.6% of the total collected samples (Figure 1).

### 3.2 RNA Sequencing and Transcriptome‐Based Subtyping

The RNA samples were profiled in three batches. Although the third batch consisted of samples that did not pass concentration cut‐off for library preparation, PCA did not show differences between the three batches, and the RNA‐Seq data was analyzed as one set (Figure S1A). Next, the impact of RNA integrity on expression profiles was assessed (Figure S1B). PCA showed that gene expression levels were affected by RIN value, and consequently, a batch correction was applied, leading to less affected expression levels (Figure S1C). After this correction, the three RNA batches did not affect the PCA results as well (Figure S1D). To check the reliability of the corrected sequencing data, the expression of Y‐linked genes was compared between males and females. As all males and only males showed Y‐linked gene expression, the batch correction was considered successful (Figure S1E).

**FIGURE 2:**
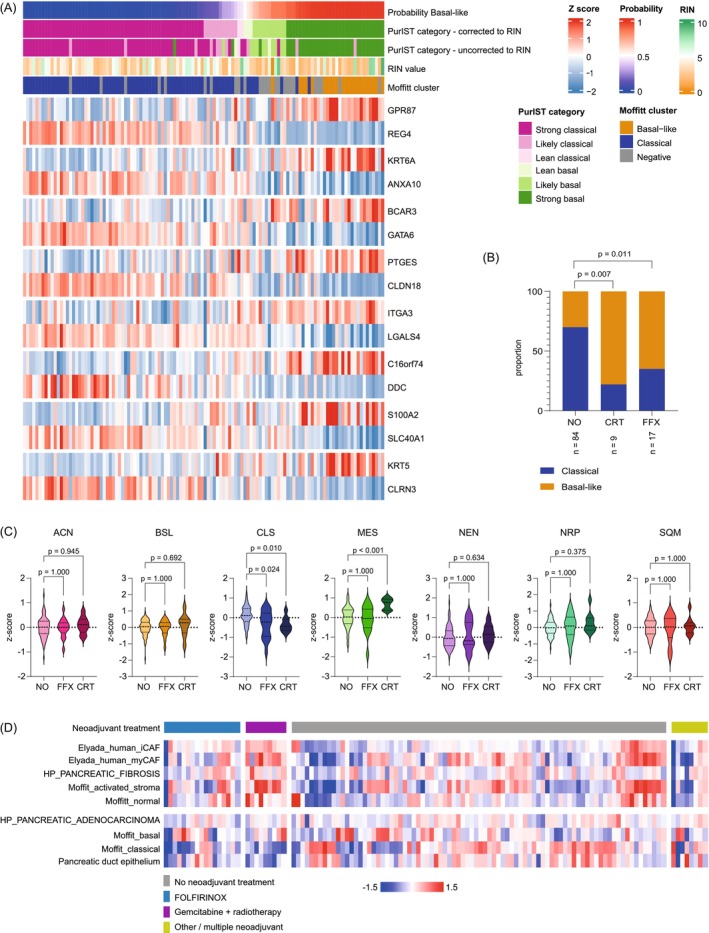
Transcriptome‐based classification of resection cohort. (A) Heatmap for the expression of PurIST classifier genes. Samples were classified according to PurIST and Moffitt classifiers. For PurIST, the RIN‐corrected and uncorrected expression levels were used. (B) Distribution of transcriptome‐based subtypes for each neo‐adjuvant treatment type. Samples excluded from the analysis: Samples that received FOLFIRINOX + radiotherapy (1), radiotherapy only (1), FOLFIRINOX + immune therapy (1) or treatments for other cancer types (4). *p* value by Fisher's exact test. (C) *Z*‐scores of Hwang single cell classifier gene signatures per neo‐adjuvant treatment type [15]. Samples excluded from the analysis: Samples that received FOLFIRINOX + radiotherapy (1), radiotherapy only (1), FOLFIRINOX + immune therapy (1) or treatments for other cancer types (4). ACN = acinar‐like, BSL = basaloid, CLS = classical, MES = mesenchymal‐like, NEN = neuroendocrine‐like, NRP = neural‐like progenitor, SQM = squamoid. Test = Kruskal–Wallis with post hoc Dunn's test. (D) *Z*‐scores for gene expression signatures derived from pancreatic stroma and epithelium. Samples were ordered by the administration of NAT with CRT. Gene expression signatures were ordered by stromal/fibroblast origin (upper panel) and epithelial/tumor cell origin (lower panel). Gene Cancer‐associated fibroblast (CAF) signatures were derived from Elyada et al. [26], pancreatic adenocarcinoma and pancreatic fibrosis gene signatures were derived from the Human Phenotype Ontology gene signatures [27], pancreatic duct epithelium signature was derived from the Human Protein Atlas [28], and the PDAC and stroma signatures from Moffitt et al. [9] were shown.

To stratify patients into prognostically relevant transcriptome‐based subgroups, the tumor samples were classified according to Moffitt and PurIST subtyping, resulting in “Classical” and “Basal‐like” subtype labels [9, 12], using the RIN value‐corrected gene expression values. Applying unsupervised clustering with the Moffitt signatures revealed a third subgroup with negative gene expression for both signatures. In this article, PurIST classification will be used for clinical and survival analyses as it is designed for use in clinical practice. However, it will be assessed whether PurIST subtyping has more prognostic accuracy than Moffitt subtyping. PurIST classification was applied to both the RIN corrected and raw set and subtype labels were compared. Eight out of 118 samples (6.8%) had a discordant subtype label between the sets. There was no statistically significant difference in RIN between the samples with discordant and consistent subtype labels (*p* = 0.25). This deviation was considered acceptable and pursued the clinical analyses using the PurIST labels based on the RIN‐corrected expression values (Figure 2A).

Next, it was assessed whether tumor subtypes were affected by tumor cell purity and stromal components. ESTIMATE scores were calculated to determine relative immune and stromal components of the samples. Within samples with low and high ESTIMATE scores, subtype proportions were equal. Furthermore, continuous ESTIMATE stromal, immune and sum scores were the same for classical and basal‐like subtypes, indicating that tumor subtypes remained stable and were not affected by tumor purity (Figure S2A–C).

### 3.3 Patient Characteristics

Clinical characteristics of included patients are shown in Table 1. During a median follow‐up period of 17.2 months, 86 patients died (72.9%) and 31 patients (26.7%) were censored. Follow‐up data was missing for one patient. For the censored patients, the median time from surgery until the last known contact date was 30.1 months (IQR: 14.1–36.6 months). The median overall survival of patients included in the RNA sequencing cohort was 17.9 months from the date of resection (95% CI 15.2–21.4 months). In the cohort, 46.6% of the included patients was female and the median age was 70.0 years. 25.3% of the patients received neoadjuvant treatment.

**TABLE 1:** Cohort overview of all patients stratified by RNA subtype.

Variable	Basal‐like (*n* = 46)	Classical (*n* = 72)	*p*	Total (*n* = 118)
Sex				
Male	27 (58.7%)	36 (50.0%)	0.463	63 (53.4%)
Female	19 (41.3%)	36 (50.0%)		55 (46.6%)
Age at surgery				
Median [Min, Max]	70.5 [52.0, 88.0]	70.0 [46.0, 85.0]	0.532	70.0 [46.0, 88.0]
Missing	0 (0%)	1 (1.4%)		1 (0.8%)
Body mass index				
Median [Min, Max]	24.3 [17.6, 32.5]	24.5 [18.1, 36.2]	0.967	24.5 [17.6, 36.2]
Missing	2 (4.3%)	3 (4.2%)		5 (4.2%)
ECOG				
0	27 (64.3%)	35 (53.8%)	0.351	62 (57.9%)
1	12 (28.6%)	27 (41.5%)		39 (36.4%)
2	3 (7.1%)	3 (4.6%)		6 (5.6%)
Missing	4	7		11
Neoadjuvant treatment				
No	27^a^ (58.7%)	60^b^ (84.5%)	**0.004**	87 (74.4%)
Gemcitabine + radiotherapy	7 (15.2%)	2 (2.8%)		9 (7.7%)
FOLFIRINOX	11 (23.9%)	9^c^ (12.7%)		20 (17.1%)
Radiotherapy	1 (2.2%)	0 (0%)		1 (0.9%)
Missing	0	1		1
Type of surgery				
Whipple	13 (28.3%)	26 (36.6%)	0.60	39 (33.3%)
Corpus—tail resection	10 (21.7%)	17 (23.9%)		27 (23.1%)
PPPD (including robot)	20 (43.5%)	26 (36.6%)		46 (39.3%)
Other	3 (6.5%)	2 (2.8%)		5 (4.23)
Missing	0	1		1
ASA score				
1	4 (9.1%)	7 (10.1%)	0.582	11 (9.7%)
2	31 (70.5%)	42 (60.9%)		73 (64.6%)
3	9 (20.5%)	20 (29.1%)		29 (25.7%)
Missing	2	3		5
Differentiation grade				
Well differentiated	3 (9.1%)	5 (9.1%)	**< 0.001**	8 (9.1%)
Moderately differentiated	12 (36.4%)	42 (76.4%)		54 (61.4%)
Poorly differentiated	18 (54.5%)	8 (14.5%)		26 (29.5%)
Missing	13	17		30
R‐status				
R0	21 (45.7%)	37 (52.1%)	0.622	58 (49.6%)
R1	25 (54.3%)	34 (47.9%)		59 (50.4%)
Missing	0	1		1
T‐status (according to UICC 8th edition)				
T1	3 (6.5%)	7 (9.9%)	0.351	10 (8.5%)
T2	35 (76.1%)	47 (66.2%)		82 (70.1%)
T3	7 (15.2%)	17 (23.9%)		24 (20.5%)
T4	1 (2.2%)	0 (0%)		1 (0.9%)
Missing	0	1		1
N‐status				
N0	11 (23.9%)	21 (29.6%)	0.549	32 (27.4%)
N1	17 (37.0%)	29 (40.8%)		46 (39.3%)
N2	18 (39.1)	21 (29.6%)		39 (33.3%)
Missing	0	1		1
Lymph node ratio cut‐off at 0.2				
0	11 (23.9%)	21 (29.2%)	0.731	32 (27.1%)
Low (< 0.2)	18 (39.1%)	28 (38.9%)		46 (39.0%)
High (> 0.2)	17 (37.0%)	22 (31.0%)		39 (33.3%)
Missing	0	1		1
Perineural invasion				
Yes	30 (78.9%)	50 (82.0%)	0.914	80 (80.8%)
No	8 (21.1%)	11 (18.0%)		19 (19.2%)
Missing	8	11		19
Angioinvasion				
Yes	24 (64.9%)	32 (53.3%)	0.365	56 (57.7%)
No	13 (35.1%)	28 (46.7%)		41 (42.3%)
Missing	9	12		21
Adjuvant chemotherapy				
Unknown/unspecified	2 (4.3%)	1 (1.4%)	0.246	3 (2.4%)
FOLFIRINOX	3 (6.5%)	10 (13.9%)	0.351	13 (11.0%)
Gemcitabine (mono)	17 (39.5%)	22 (31.0%)	0.466	39 (34.2%)
Gemcitabine + capecitabin	3 (6.5%)	14 (19.4%)	0.0954	17 (14.4%)
None	21 (45.7%)	25 (34.7%)	0.237	46 (39.0%)
Registration of disease recurrence (local and distant) in follow‐up				
Yes	35 (76.1%)	46 (63.9%)	0.2342	81 (68.6%)
No	11 (23.9%)	26 (36.1%)		37 (31.4%)
Distant metastasis in follow‐up				
Yes	32 (71.1%)	38 (53.5%)	0.0906	70 (60.3%)
No	13 (28.9%)	33 (46.5%)		46 (39.7%)
Missing	1	1		2
Systemic treatment for disease recurrence (local and distant) in follow‐up				
Yes	5 (11.6%)	14 (20.9%)	0.319	19 (17.3%)
No	38 (88.4%)	53 (79.1%)		91 (82.7%)
Missing	3	5		8

*Note:* Information was retrieved from patients' reports. Some patients were referred to other hospitals for adjuvant treatment, and the start and type of treatment were not confirmed. Proportions were presented and tested excluding the missing cases. *p*‐values of 0.05 or lower were provided in bold font.

Abbreviations: ASA, American Society of Anesthesiology; ECOG, Eastern Cooperative Oncology Group.

^a^ One patient received hormonal therapy and one patient received neoadjuvant radiotherapy, both for other cancer types.

^b^ One patient received radiotherapy for another cancer type.

^c^ One patient received FOLFIRINOX with radiotherapy, one patient received FOLFIRINOX with immunomodulatory therapy and one patient had radiotherapy for another cancer type in treatment history.

### 3.4 Transcriptomic Analysis of Neo‐Adjuvant Treated Samples

No baseline differences existed between the classical and basal‐like transcriptome‐based subgroups for sex, age, BMI, performance status and American Society of Anesthesiology (ASA) score (Table 1). However, differentiation grade differed significantly between the subtypes. A poorer differentiation was observed in basal‐like tumors. The basal‐like subtype was also statistically significantly associated to NAT, although the subgroups were relatively small. Significantly more patients who received any type of NAT had a basal‐like tumor (19/30, 63.3%) compared to NAT‐naïve patients (27/87, 31.0%) (*p* = 0.004). When studying specific neoadjuvant regimens, both the samples treated with NAT with gemcitabine and radiotherapy (CRT) and those pre‐treated with FOLFIRINOX were enriched for the basal‐like subtype (CRT: basal‐like in 7/9 tumors; *p* = 0.007, FOLFIRINOX: basal‐like in 11/17 tumors; *p* = 0.011) (Figure 2B).

To study the effect of NAT on gene expression in more detail, the top differentially expressed genes of the tumor cell‐intrinsic single cell‐derived signatures (Hwang et al.) were applied to the gene expression data (Figure 2C). The basaloid (*p* = 0.64) and neural‐like progenitor (*p* = 0.37) signatures were both not significantly affected by NAT. Classical‐like signaling was significantly decreased (*p* = 0.0018), suggesting that the higher prevalence of basal‐like subtype in the NAT‐treated subgroup was caused by a decrease of classical‐like tumor cells following NAT. CRT‐pretreated tumors had a significantly increased mesenchymal‐like gene signature (*p* < 0.001). Although this gene signature was derived from tumor cells [15], it was explored whether this upregulation in bulk RNA was indeed tumor cell‐intrinsic or irradiation‐induced fibrosis of the stroma [29, 30]. When comparing epithelial‐, fibrosis‐ and fibroblast‐derived gene signatures between samples with and without CRT, the stromal pathways were elevated after CRT (Figure 2D). This was confirmed by a significant increase of the ESTIMATE stromal score upon CRT compared to no NAT (*p* < 0.001) and NAT with FFX (*p* = 0.009) (Figure S2C).

To gain more insight into NAT‐induced gene expression, gene signatures were established for samples treated with either NAT‐FFX or NAT‐CRT (Section 2). Next, the expression of these gene signatures was correlated with expression for several classifier‐ and literature‐derived gene signatures. In line with the abovementioned results, the NAT‐CRT gene expression signature correlated positively with gene signatures for mesenchymal PDAC subtypes and fibroblasts. Negative correlations were found for classical PDAC subtype gene signatures (Table S1A). For the NAT‐FFX gene signature, only weak negative correlations were found for classical PDAC subtype gene signatures. Strongest correlations were found with the Hwang single cell‐derived neuroendocrine and neural‐like progenitor subtype gene signatures (Table S1A). To add more background, the NAT‐FFX signature was correlated to the Broad Institute's Hallmark and Cell type gene signatures, and strongest correlations were found with gene signatures for neural cell types (Table S1B+C).

### 3.5 Survival Analysis

**FIGURE 3:**
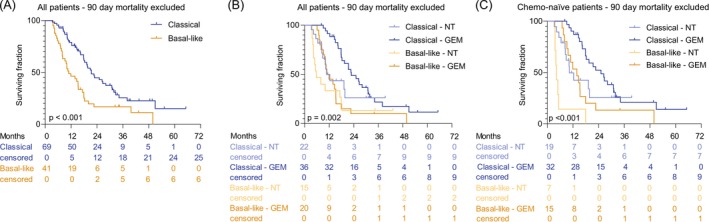
Overall survival after surgery for transcriptome‐based subtypes. Patients who died or were censored within 90 days were excluded. Overall, the Log‐Rank test is indicated. Numbers of patients at risk and censored patients are provided. (A) OS for the complete cohort (*n* = 110) per RNA subtype. (B) OS for patients treated with adjuvant gemcitabine (with or without capecitabine, *n* = 56) compared to patients without adjuvant treatment (*n* = 37). (C) OS for patients who did not receive NAT treated with adjuvant gemcitabine (with or without capecitabine, *n* = 47) compared to patients without adjuvant treatment (*n* = 26).

In the study cohort, patients with a classical tumor had a significantly longer OS than patients with a basal‐like tumor (21.4 (95% CI: 18.2–31.0) vs. 10.9 (95% CI: 9.2–17.2) months, *p* < 0.001, Figure 3A). Most patients (58.5%, 69/118) received adjuvant chemotherapy after resection, of whom 81.2% (56/69) received gemcitabine (with or without capecitabine) and 18.8% (13/69) received FOLFIRINOX. Patients in this cohort received capecitabine since 2017 and FOLFIRINOX since 2018, and neoadjuvant treatments were administered as well (Figure S3A–C). There was no statistically significant association between adjuvant treatment type and Eastern Cooperative Oncology Group (ECOG) score (*p* = 0.31). Significant survival differences were observed between adjuvant treatment type, as median OS was 10.2 (95% CI: 8.3–19.2) months in patients without adjuvant treatment, 17.9 (95% CI: 15.2–25.1) months in gemcitabine‐treated patients and 39.8 (95% CI: 32.7–NA) months in FOLFIRINOX‐treated patients (overall *p* = 0.002, Figure S3D). Patients treated with NAT did not show longer survival rates than NAT‐naïve patients (within adjuvant receiving patients: 21.4 (95% CI: 13.9–NA) months in patient receiving NAT vs. 20.9 (95% CI: 17.8–30.9) months in patients without NAT, *p* = 0.71; and within patients receiving no adjuvant treatment: 17.2 (95% CI: 10.0–NA) months in patients receiving NAT vs. 8.8 (95% CI: 6.4–19.2) months in patients without NAT, *p* = 0.36) (see Figure S3E for OS curves per treatment type). Consequently, the association between survival and transcriptome‐based subgroups within each treatment group was assessed. Given that a quarter of the patients received NAT, which could potentially induce a subtype shift or resistance for systemic treatment post‐surgery [31], survival analyses were performed in the whole cohort as well as in the selection of NAT‐naïve patients who underwent immediate surgery.

In both transcriptome‐based classical and basal‐like subgroups, a similar proportion of patients received adjuvant chemotherapy. The median duration of adjuvant treatment was similar in both groups (4.0 months in patients with basal‐like tumors and 4.9 months in patients with classical tumors, *p* = 0.87). The group of patients receiving adjuvant FOLFIRINOX was small (*n* = 13), and therefore, survival differences between transcriptome‐based subtypes in specifically adjuvant FOLFIRINOX‐treated patients were not studied in more detail or corrected for clinical variables. In the cohort of patients treated with adjuvant gemcitabine (with or without capecitabine), those with a classical tumor had longer OS compared to those with a basal‐like tumor (median OS = 22.8 (95% CI: 18.2–31.8) vs. 11.4 (95% CI: 9.7–15.3) months; *p* < 0.001, Figure 3B). However, for both tumor subtypes, the OS was not significantly longer for adjuvant gemcitabine‐treated patients compared to patients without adjuvant treatment, although this might be caused by the small sample sizes (in patients with classical tumors: median OS = 22.8 (95% CI: 18.2–31.8) months with adjuvant gemcitabine vs. 10.7 (95% CI: 8.1–NA) months without adjuvant treatment; *p* = 0.061, in patients with basal‐like tumors: median OS = 11.4 (95% CI: 9.7–15.3) months with adjuvant gemcitabine vs. 5.4 (95% CI: 4.3–18.2) months without adjuvant treatment; *p* = 0.52).

Among the NAT‐naïve patients, OS significantly differed between gemcitabine‐treated classical and basal‐like tumors (median OS = 25.1 (95% CI: 18.2–31.8) vs. 13.8 (95% CI: 9.7–22.6) months; *p* = 0.005, Figure 3C). In patients with a basal‐like tumor, there was a significant difference in OS between patients receiving adjuvant gemcitabine and patients receiving no adjuvant treatment (median OS = 13.8 (95% CI: 9.7–22.6) months with adjuvant gemcitabine vs. 4.3 (95% CI: 3.6–NA) months without adjuvant treatment; *p* = 0.006). In patients with a classical tumor, the survival difference between these groups was not significant (median OS = 25.1 (95% CI: 18.2–31.8) months with adjuvant gemcitabine vs. 10.3 (95% CI: 8.4–NA) months without adjuvant treatment; *p* = 0.061). Similar patterns were observed for DFS (Figure S4).

### 3.6 Multivariable Cox Regression Analysis

To study whether transcriptome‐based subtyping improves prognostic modeling with clinical and pathological variables, multivariable correction was performed, and prognostic model performance was assessed after addition of transcriptome‐based subtyping. In both the complete cohort and the selection of NAT‐naïve patients, the classical subtype was significantly associated with longer survival after multivariable correction (complete cohort: hazard ratio (HR) = 0.38 (95% CI = 0.21–0.71); *p* = 0.002, NAT‐naïve cohort: HR = 0.30 (95% CI = 0.14–0.63); *p* = 0.001). The addition of transcriptome‐based subtyping to the Amsterdam model improved the model significantly (complete cohort: *p* = 0.002, NAT‐naïve cohort: *p* = 0.001). The prognostic model including transcriptome‐based subtyping had a lower AIC and BIC compared to the model without subtyping, in both the whole cohort and the NAT‐naïve cohort (whole cohort: AIC = 432 and BIC = 451 with subtyping vs. AIC = 439 and BIC = 457 without subtyping; NAT‐naïve cohort: AIC = 352 and BIC = 368 with subtyping vs. AIC = 360 and BIC = 374 without subtyping) (Tables 2 and 3). In the prognostic model, differentiation grade and adjuvant chemotherapy were independently and statistically significantly associated with overall survival. To confirm that the transcriptome‐based subtypes had prognostic value in patients treated with adjuvant gemcitabine, the same analysis was conducted in a subgroup of patients treated with adjuvant gemcitabine, and this yielded similar results: HR for classical subtype = 0.22 (95% CI = 0.09–0.49); *p* < 0.001 (Table S2). Next, it was studied whether assessment of the Moffitt subtypes improved prognostic modeling in the same way as addition of PurIST subtyping. However, applying the same multivariable model using Moffitt subtypes instead of PurIST subtypes did not yield a significant relation between subtype and OS (Table S3). Furthermore, the AIC and BIC were better for the model using PurIST subtypes (AIC = 432, BIC = 451) than the Moffitt subtypes (AIC = 441, BIC = 463).

**TABLE 2:** Uni‐ and multivariable Cox regression analysis results for all patients.

	Univariable		Multivariable	
	HR (95% CI)	*p*	HR (95% CI)	*p*
LNR = 0	Reference		Reference	
LNR > 0 & LNR < 0.2	1.20 (0.67–2.12)	0.542	1.20 (0.60–2.40)	0.603
LNR > 0.2	1.72 (0.95–3.08)	0.071	1.34 (0.67–2.69)	0.406
Well differentiated	Reference		Reference	
Moderately differentiated	2.66 (0.95–7.48)	0.063	4.19 (1.33–13.16)	**0.014**
Poorly differentiated	6.20 (2.09–18.37)	**0.001**	7.59 (2.47–23.31)	**< 0.001**
R0 resection	Reference		Reference	
R1 resection	1.30 (0.83–2.02)	0.253	1.02 (0.59–1.73)	0.956
PurIST basal‐like	Reference		Reference	
PuriST classical	0.47 (0.30–0.74)	**0.001**	0.38 (0.21–0.71)	**0.002**
Adjuvant chemotherapy	Reference		Reference	
No adjuvant chemotherapy (tgroup 1)	6.82 (2.66–17.47)	**< 0.001**	11.04 (3.78–32.21)	**< 0.001**
No adjuvant chemotherapy (tgroup 2)	1.61 (0.57–4.59)	0.369	2.59 (0.79–8.43)	0.115
No adjuvant chemotherapy (tgroup 3)	0.71 (0.27–1.81)	0.469	0.33 (0.07–1.47)	0.145

*Note:* The multivariable analysis was a complete‐case analysis and patients who died or were censored within 90 days were excluded (*N* = 82). The addition of PurIST subtype to the prognostic model improved the model significantly (*p* = 0.002). The effect of adjuvant chemotherapy on overall survival was time‐varying. *p*‐values of 0.05 and lower were provided in bold font. Tgroup 1 = 0–275 days, tgroup 2 = 275–440 days, tgroup 3 = 450 days or longer.

Abbreviations: 95% CI, 95% confidence interval; HR, hazard ratio; LNR, lymph node ratio.

**TABLE 3:** Uni‐ and multivariable Cox regression analysis results for NAT‐naïve patients.

	Univariable		Multivariable	
	HR (95% CI)	*p*	HR (95% CI)	*p*
LNR = 0	Reference		Reference	
LNR > 0 & LNR < 0.2	1.19 (0.59–2.40)	0.632	1.84 (0.81–4.16)	0.145
LNR > 0.2	1.79 (0.90–3.57)	0.098	1.82 (0.83–4.02)	0.137
Well differentiated	Reference		Reference	
Moderately differentiated	2.88 (0.88–9.41)	0.079	4.55 (1.26–16.41)	**0.021**
Poorly differentiated	6.60 (1.91–22.77)	**0.003**	6.40 (1.79–22.92)	**0.004**
R0 resection	Reference		Reference	
R1 resection	1.32 (0.80–2.19)	0.279	1.18 (0.67–2.08)	0.576
PurIST subtype basal‐like	Reference		Reference	
PurIST subtype classical	0.37 (0.22–0.64)	**< 0.001**	0.30 (0.14–0.63)	**0.001**
Adjuvant chemotherapy	Reference		Reference	
No adjuvant chemotherapy (tgroup = 1)	9.91 (3.22–30.50)	**< 0.001**	19.6 (5.25–73.13)	**< 0.001**
No adjuvant chemotherapy (tgroup = 2)	1.01 (0.43–2.42)	0.976	1.96 (0.70–5.47)	0.197

*Note:* The multivariable analysis was a complete‐case analysis and patients who died or were censored within 90 days were excluded (*N* = 72). The addition of PurIST subtype to the prognostic model improved the model significantly (*p* = 0.001). The effect of adjuvant treatment on overall survival was time‐dependent. *p*‐values of 0.05 and lower were provided in bold font. Tgroup 1 = 0–270 days, tgroup 2 = 270 days or longer.

Abbreviations: 95% CI, 95% confidence interval; HR, hazard ratio; LNR, lymph node ratio.

Given the substantial part of patients for which differentiation grade was missing (30/118, 25.4%), mainly those receiving NAT (18/30, 60.0%), analyses were repeated including these patients (Section 2, Tables S4–S6). These models yielded similar results as reflected by significant hazard ratios for the same variables as in the complete‐case models. Thus, in summary, the transcriptome‐based classical subtype, better differentiation grade (when this variable was available) and adjuvant chemotherapy were all independently and significantly prognostic for a longer survival after resection.

## 4 Discussion

This research showed that integrating transcriptome‐based subtyping improved prognostic modeling for overall survival after PDAC resection and adjuvant treatment with gemcitabine, compared to relying solely on pathological variables. This highlights the potential of transcriptomic profiling to uncover molecular heterogeneity that remains undetected by traditional pathology. In daily clinical practice, the ability to refine prognostic accuracy could enable patients and clinicians to make better‐informed treatment decisions, for example, choosing for or refraining from aggressive adjuvant therapies based on prognostic information. Furthermore, given its strong prognostic power, transcriptome‐based subtyping could be a relevant factor for stratification in clinical trials.

While others already showed that transcriptome‐based subtyping was prognostic in resected pancreatic cancer independent of pathological and clinical characteristics [13, 32], this study was the first to show that the accuracy of prognostic modeling significantly improves when transcriptome‐based subtyping was added to prognostic models based on clinical characteristics, such as the internationally validated Amsterdam prognostic model [24]. Our study included patients who were treated with and without adjuvant chemotherapy, enabling us to study OS after adjuvant gemcitabine using the PurIST classifier. This classifier utilizes the gene expression of a small number of genes [12] and will likely be more easy to implement in clinical practice than other classification tools that were significantly associated with chemotherapy response and use higher numbers of signature genes [33, 34]. The high number of collaborating hospitals to our study makes this cohort unique compared to studies conducted in single‐center sample archives and improves the representativity of the cohort.

Adjuvant gemcitabine‐treated patients with classical tumors exhibited longer overall survival compared to those with basal‐like tumors. While the Kaplan Meier curves did not show a significant longer OS for patients treated with adjuvant gemcitabine versus no treatment for both subtypes, the Cox regression analysis showed that adjuvant treatment remained an independent prognostic factor for survival, suggesting that both classical and basal subtypes benefit from adjuvant chemotherapy. The effect of adjuvant chemotherapy varied over time, showing significance only in the earlier time intervals. Kaplan Meier curves confirmed this, displaying a longer median OS for gemcitabine‐treated versus non‐treated patients but with crossing survival curves at the later time points. This time‐varying effect of adjuvant treatment has not been observed before in trials investigating the currently‐used adjuvant chemotherapy [5, 35, 36]. Adjuvant treatment aims to prevent the outgrowth of microscopic, undetected metastases that may already be present during surgery for the primary tumor or microscopic tumor residue after surgery. We hypothesize that adjuvant treatment often delays the progression of these micrometastases into detectable recurrences, or selects for chemoresistant clones, rather than eliminating all residual cancer cells. This could explain the prognostic effect for adjuvant treatment at the early time points, which diminishes at later time points. The classical tumors recur, or switch to a more chemoresistant phenotype at a slower pace than the basal‐like tumors irrespective of adjuvant chemotherapy given (Figure S4). Therefore, the poor prognosis of the basal‐like subtype seems to be independent of adjuvant chemotherapy response, which is confirmed by the significant *p*‐values in the multivariable model for both variables.

Compared to what was found in other subtyping efforts, the proportion of basal‐like tumors in our cohort was relatively high, typically in NAT‐treated samples, although our groups were relatively small [12, 13]. There is currently no consensus regarding the impact of NAT on tumor subtypes and the potential development of subsequent chemotherapy resistance. In a single‐cell RNA sequencing analysis of freshly taken PDAC samples, no shift towards more basal‐like cells was observed upon chemotherapy, which was in line with our analysis using single‐cell gene signatures (Figure 2C) [15, 37]. However, a study that included bulk‐resected PDAC specimens observed an increased basal‐like RNA subtype after NAT [14]. A surrogate immunohistochemistry (IHC) marker replacing RNA expression data for the classical subtype is high expression of GATA6 and a surrogate marker for the basal‐like subtype is keratin‐5 (CK5) [34, 38]. One study investigating the effect of NAT, mainly gemcitabine‐based chemotherapy regimens, observed a decrease of both GATA6 and CK5 expression after NAT [38]. In another analysis of NAT‐treated and NAT‐naïve resection specimens, it was observed that high GATA6 expression was only significantly associated with survival in NAT‐naïve patients and not in the patients who received neoadjuvant CRT [39]. In contrast to single cell‐derived basal‐like signatures, mesenchymal signatures were significantly increased in CRT‐treated samples (Figure 2C). Elevation of fibroblast‐derived pathways after CRT, as shown in Figure 2D, might indicate that the upregulation of this mesenchymal signatures may reflect a change in the tumor microenvironment rather than in the tumor cells. This might affect bulk sequencing results. Importantly, the NAT‐affected bulk sequencing results might reflect either tumor‐intrinsic changes, microenvironmental modeling due to radiation, or both. In contrast to the study of Hwang et al. [15], no significant change in the neural‐like progenitor signature was observed. This effect might be kept hidden in the bulk sequencing results because of the NAT‐induced changes in the more abundant stromal compartment. Of note, we found a significant correlation between FOLFIRINOX‐upregulated gene expression and neural‐like progenitor gene expression (Table S1). This correlation does not imply that the neural‐like progenitor subtype itself increases, as the two signatures share almost no overlapping genes. It does, however, suggest a possible broader relationship between neural‐like expression patterns and FOLFIRINOX treatment. Lastly, the samples for RNA analysis were selected based on tumor cell percentage. The residual tumor cells in the resection specimens of NAT‐treated patients might be more resistant to chemotherapy than NAT‐naïve tumor cells. A higher number of residual tumor cells after NAT was significantly associated with poor survival [40]. These findings suggest that RNA‐based subtyping in NAT‐treated samples might not work or lead to biased results. Implementation of RNA‐based subtyping on EUS‐guided biopsies that were acquired before NAT might solve this.

The results of this study should be considered in light of some limitations. First, in the adjuvant setting, both FOLFIRINOX and gemcitabine + capecitabine have proven to lead to a more prolonged OS than gemcitabine monotherapy [6, 36]. The present cohort lacks sufficient numbers of patients receiving these treatment combinations to assess separately in a survival analysis. However, studies in the metastatic setting show a survival benefit for classical over basal‐like tumors with FOLFIRINOX chemotherapy, suggesting that a similar effect could occur for FOLFIRINOX in the adjuvant setting [34, 41, 42]. Second, while the use of a binary subtyping method facilitates patient stratification and is more feasible in clinical practice, it might oversimplify classification. PDACs often contain both classical and basal‐like cells (“mixed” tumors) [37, 41] and even cells that are in an intermediate stage (“intermediate” cells) [43, 44]. The presence of intermediate or basal‐like cells might induce a quick development of chemoresistance, even if the tumor was originally classified as classical. Third, only a quarter of the included samples had sufficient tumor cellularity for RNA sequencing. Our experimental pipeline required a minimal pathologist‐assessed tumor cellularity of 30%. However, pancreatic tumors are characterized by desmoplastic stroma and low tumor cellularity compared to other cancer types, and tumor tissue is hard to distinguish macroscopically from pancreatitis. Thus, most samples do not meet this requirement [45, 46], hindering the implementation of this technique in diagnostic pipelines. One way to optimize sampling would be the implementation of laser microdissection to increase tumor cellularity [47, 48], although this method might not be feasible due to the long time needed to execute the protocol for one sample [49]. Probably a more feasible way to handle the low tumor cellularity and the abovementioned coexistence of classical and basal‐like cells in one tumor, is the implementation of IHC‐based subtyping. Subtyping based on IHC markers, like the classical marker GATA6 and cytokeratins as basal‐like markers, will provide a better insight into intra‐tumor heterogeneity and spatial variance compared to bulk sequencing. Another method to improve classification in low‐purity tumors might be the subtyping of fibroblasts, which are highly abundant and have prognostic value [50]. Third, this relative high tumor cellularity requirement might affect the representativity of the study's survival results. Although we showed in previous work that tumor cell percentage of a metastatic biopsy was not related to OS [51], others showed that a higher tumor cellularity is significantly associated with a shorter survival after resection in NAT‐treated and NAT‐naïve pancreatic cancer patients [40, 52, 53]. Therefore, selection for high tumor cell percentage might select for non‐responders and chemoresistant tumors, in particular in NAT‐treated patients. In NAT‐naïve patients, the presence of residual tumor cells might be more important for OS than the actual number of residual tumor cells. Lastly, our group of NAT patients was too small (*n* = 30) to validate the effect of NAT on subtype‐based survival prediction. Study material obtained from larger cohorts, including pre‐NAT biopsies and post‐NAT resection samples, should be investigated to confirm whether NAT induces a shift in subtype and whether pre‐treated basal‐like tumors respond differently to chemotherapy than NAT‐naïve basal‐like tumors.

In conclusion, transcriptome‐based subtyping is independently prognostic for overall survival after resection of PDAC in patients with and without adjuvant treatment. Consequently, transcriptome‐based subtyping could provide additional prognostic information to patients and should be considered as a stratification factor in clinical trials. Furthermore, subtyping of NAT‐treated resection material should be done cautiously if there is no consensus regarding the pre‐treatment effect on subtype and subsequent chemoresistance.

## Author Contributions

**Marjolein F. Lansbergen:** conceptualization, methodology, investigation, formal analysis, data curation, writing – original draft, visualization, project administration. **Vincent R. Lanting:** investigation, data curation, writing – review and editing. **Paul Manoukian:** investigation, data curation, writing – review and editing. **Marc G. Besselink:** resources, writing – review and editing. **Geert Kazemier:** resources, writing – review and editing. **Ignace H. J. T. de Hingh:** writing – review and editing, resources. **Mike S. L. Liem:** writing – review and editing, resources. **Casper H. J. van Eijck:** writing – review and editing, resources. **Erwin van der Harst:** writing – review and editing, resources. **Vincent E. de Meijer:** writing – review and editing, resources. **Ronald M. van Dam:** writing – review and editing, resources. **Martijn W. J. Stommel:** writing – review and editing, resources. **Jan Koster:** software, resources, data curation, writing – review and editing. **Michael W. T. Tanck:** methodology, writing – review and editing. **Arantza Fariña Sarasqueta:** investigation, writing – review and editing. **Joanne Verheij:** investigation, writing – review and editing. **Frederike Dijk:** resources, writing – review and editing. **Johanna W. Wilmink:** writing – review and editing, resources. **Maarten F. Bijlsma:** resources, conceptualization, writing – review and editing, supervision, funding acquisition. **Hanneke W. M. van Laarhoven:** conceptualization, resources, writing – review and editing, supervision, funding acquisition.

## Funding

This research is supported by funding to H.W.M.L. and M.F.B. from the Dutch ZonMw “Goed Gebruik Geneesmiddelen” program, grant number 848101012. The funding body had no role in the design of the study, the collection, analysis and interpretation of the data and the writing of the manuscript.

## Ethics Statement

Patients gave written informed consent for participation in either the Dutch Pancreas biobank (METC 2014_180) or the VUmc BioHPB (METC 2022.0068). In all participating hospitals a medical ethical committee, biobank testing committee, or a local feasibility advisory committee approved the release of samples from the biobank and the collection of clinical data.

## Conflicts of Interest

M.F.B.: Research funding: Celgene, Frame Therapeutics, Lead Pharma. Consultant/advisory role: Servier, Olympus, Wholomics. H.W.M.L.: Consultant/advisory role: AMGEM, Amphera, Astellas, AstraZeneca, Beigene, BMS, Boehringer, Daiichy, MSD, MyeloidTx. Speaker role: AstraZeneca, BMS, Congress Care, Daiichy, Medtalks, Uitgeverij JAAP, Travel Congress Management. Research funding and/or medication/material supply: AMGEM, Auristone, BMS, Incyte, Merck, MyeloidTx, ORCA, Servier. J.W.W.: Research funding and/or medication supply: Servier, MSD, Nordic. Consultant/advisory role: MSD, Servier, Astra Zeneca, none related to the manuscript. R.M.D.: Research funding; Abbot Labotories, Guerbet. Consultant role: Barco, paid to Maastricht University. The other authors declare no conflicts of interest.

## Supporting information


**Table S1:** Neoadjuvant treatment‐related gene expression signatures correlated to literature‐derived gene signatures.

**Table S2:** Uni‐ and multivariable Cox regression analysis results for adjuvant gemcitabine‐treated patients.
**Table S3:** Multivariable Cox regression analysis results for all patients.
**Table S4:** Uni‐ and multivariable Cox regression analysis results for all patients, including missing differentiation grade.
**Table S5:** Uni‐ and multivariable Cox regression analysis results for NAT‐naïve patients, including missing differentiation grade.
**Table S6:** Uni‐ and multivariable Cox regression analysis results for adjuvant gemcitabine‐treated patients, including missing differentiation grade.
**Figure S1:** Effect of RIN‐value and RIN‐based correction on expression levels.
**Figure S2:** ESTIMATE scores for PurIST subtypes. Low ESTIMATE sum scores indicate a high tumor purity.
**Figure S3:** Neoadjuvant and adjuvant treatment regimens in the RNA cohort.
**Figure S4:** Disease‐free survival after surgery for transcriptome‐based subtypes.

**Table S7:** RNA‐sequencing quality statistics.

## Data Availability

Normalized count data is available in GEO under accession number GSE310252. The raw sequencing data is available on the Hartwig Medical Foundation Database under restricted access and is categorized as “pancreatic cancer, resectable”. Requests for the raw sequencing data and clinical variables need to be made through a Data Access Request with the Hartwig Medical Foundation https://www.hartwigmedicalfoundation.nl/en/data/data‐access‐request/. Other data that support the findings of this study are available from the corresponding author upon request.
