# Supplementary material for: Transcriptome‐Based Classification of Resected Pancreatic Ductal Adenocarcinoma Enhances Prognostic Modelling Accuracy of Overall Survival Following Adjuvant Treatment
Source: Int J Cancer. 2026 Apr 27;159(5):1322–36. doi: 10.1002/ijc.70519 (PMC13340967; doi:10.1002/ijc.70519)
Supplement: Supplementary file 2 — Table S2: Uni‐ and multivariable Cox regression analysis results for adjuvant gemcitabine‐treated patients. Table S3: Multivariable Cox regression analysis results for all patients. Table S4: Uni‐ and multivariable Cox regression analysis results for all patients, including missing differentiation grade. Table S5: Uni‐ and multivariable Cox regression analysis results for NAT‐naïve patients, including missing differentiation grade. Table S6: Uni‐ and multivariable Cox regression analysis results for adjuvant gemcitabine‐treated patients, including missing differentiation grade. Figure S1: Effect of RIN‐value and RIN‐based correction on expression levels. Figure S2: ESTIMATE scores for PurIST subtypes. Low ESTIMATE sum scores indicate a high tumor purity. Figure S3: Neoadjuvant and adjuvant treatment regimens in the RNA cohort. Figure S4: Disease‐free survival after surgery for transcriptome‐based subtypes. [file IJC-159-1322-s002.pdf]

SUPPLEMENTARY MATERIAL FOR

Transcriptome-based classification of resected pancreatic ductal adenocarcinoma enhances prognostic modelling accuracy of overall survival following adjuvant treatment

Marjolein F. Lansbergen, Vincent R. Lanting, Paul Manoukian, Marc G. Besselink, Geert Kazemier, Ignace H.J.T. de Hingh, Mike S.L. Liem, Casper H.J. van Eijck, Erwin van der Harst, Vincent E. de Meijer, Ronald M. van Dam, Martijn W.J. Stommel, Jan Koster, Michael W.T. Tanck, Arantza Fariña, Joanne Verheij, Frederike Dijk, Johanna W. Wilmink, Maarten F. Bijlsma, Hanneke W.M. van Laarhoven for the Dutch Pancreatic Cancer Group.

TABLE OF CONTENTS

Supplementary Tables 1–7
Supplementary Figures 1–4

SUPPLEMENTARY TABLES

Supplemental Table 1: Neoadjuvant treatment-related gene expression signatures correlated to literature-derived gene signatures. R-, t- and p-values were provided. Df = degrees of freedom. A: Neoadjuvant treatment-related gene signatures correlated to PDAC-classifier gene signatures. B: Neoadjuvant FOLFIRINOX-related gene signature correlated to Broad Institute’s C8 Cell types gene signatures. C: Neoadjuvant FOLFIRINOX-related gene signature correlated to Broad Institute’s Hallmark gene signatures. Uploaded as an Excel file.

Supplemental Table 2: Uni- and multivariable Cox regression analysis results for adjuvant gemcitabine-treated patients. The multivariable analysis was a complete-case analysis and patients who died or were censored within 90 days were excluded (N = 49). The addition of PurIST subtype to the prognostic model again improved the model (p = 0.0002). LNR = lymph node ratio. HR = hazard ratio. 95% C.I. = 95% confidence interval.

	**Univariable**		**Multivariable**	
	**HR (95% C.I.)**	**p-value**	**HR (95% C.I.)**	**p-value**
LNR = 0	Reference		Reference	
LNR > 0 & LNR < 0.2	1.25 (0.59 - 2.63)	0.563	0.74 (0.31 - 1.75)	0.496
LNR > 0.2	1.59 (0.72 - 3.50)	0.248	0.69 (0.28 - 1.72)	0.423
Well differentiated	Reference		Reference	
Moderately differentiated	2.19 (0.65 - 7.31)	0.204	7.92 (1.65 - 38.05)	**0.010**
Poorly differentiated	5.74 (1.63 - 20.21)	**0.006**	14.21 (3.16 - 63.85)	**0.001**
R0 resection	Reference		Reference	
R1 resection	0.88 (0.49 - 1.57)	0.667	0.72 (0.35 - 1.45)	0.351
PurIST Basal-like	Reference		Reference	
PurIST Classical	0.33 (0.18 - 0.60)	**< 0.001**	0.20 (0.09 - 0.48)	**< 0.001**

Supplemental Table 3: Multivariable Cox regression analysis results for all patients. The multivariable analysis was a complete-case analysis and patients who died or were censored within 90 days were excluded (N = 82). The effect of adjuvant chemotherapy on overall survival was time-varying. Tgroup 1 = 0 – 275 days, tgroup 2 = 275 – 440 days, tgroup 3 = 450 days or longer. LNR = lymph node ratio. HR = hazard ratio. 95% C.I. = 95% confidence interval.

	**HR (95% C.I.)**	**p-value**
LNR = 0	Reference	
LNR > 0 & LNR < 0.2	1.32 (0.66 – 2.64)	0.437
LNR > 0.2	1.32 (0.67 – 2.62)	0.420
Well differentiated	Reference	
Moderately differentiated	2.68 (0.89 – 8.02)	0.078
Poorly differentiated	6.00 (1.96 – 18.36)	**0.002**
R0 resection	Reference	
R1 resection	1.13 (0.67 – 1.93)	0.645
Moffitt subtype Classical	Reference	
Moffitt subtype Basal-like	1.54 (0.68 – 3.50)	0.305
Moffitt subtype Negative	1.66 (0.83 – 3.34)	0.152
Adjuvant chemotherapy	Reference	
No adjuvant treatment (tgroup = 1)	10.17 (3.48 – 29.73)	**<0.001**
No adjuvant treatment (tgroup = 2)	2.65 (0.81 – 8.64)	0.106
No adjuvant treatment (tgroup = 3)	0.42 (0.09 – 1.88)	0.256

Supplemental Table 4: Uni- and multivariable Cox regression analysis results for all patients, including missing differentiation grade. The multivariable analysis was a complete-case analysis, except for differentiation grade. If differentiation grade was missing, this was treated as an extra category of differentiation grade. Patients who died or were censored within 90 days were excluded (N = 109). The addition of PurIST subtype to the prognostic model improved the model significantly (p = 0.001). The effect of adjuvant chemotherapy on overall survival was time-varying. Tgroup 1 = 0 – 275 days, tgroup 2 = 275 – 440 days, tgroup 3 = 450 days or longer. LNR = lymph node ratio. HR = hazard ratio. 95% C.I. = 95% confidence interval.

	**Univariable**		**Multivariable**	
	**HR (95% C.I.)**	**p-value**	**HR (95% C.I.)**	**p-value**
LNR = 0	Reference		Reference	
LNR > 0 & LNR < 0.2	1.20 (0.67 - 2.12)	0.542	1.38 (0.74 - 2.58)	0.306
LNR > 0.2	1.72 (0.95 - 3.08)	0.071	1.56 (0.82 - 2.99)	0.177
Well differentiated	Reference		Reference	
Differentiation grade missing	1.76 (0.58 – 5.36)	0.321	1.86 (0.57 - 6.02)	0.300
Moderately differentiated	2.62 (0.93 – 7.35)	0.068	3.66 (1.21 - 11.05)	**0.022**
Poorly differentiated	5.81 (1.97 – 17.12)	**0.001**	6.96 (2.30 - 21.05)	**0.001**
R0 resection	Reference		Reference	
R1 resection	1.30 (0.83 - 2.02)	0.253	1.10 (0.67 - 1.80)	0.707
PurIST subtype Basal-like	Reference		Reference	
PurIST subtype Classical	0.47 (0.30 - 0.74)	**0.001**	0.42 (0.25 - 0.71)	**0.001**
Adjuvant chemotherapy	Reference		Reference	
No adjuvant chemoth. (tgroup = 1)	6.82 (2.66 - 17.47)	**< 0.001**	9.96 (3.78 - 26.25)	**< 0.001**
No adjuvant chemoth. (tgroup = 2)	1.61 (0.57 - 4.59)	0.369	2.10 (0.72 - 6.10)	0.174
No adjuvant chemoth. (tgroup = 3)	0.71 (0.27 - 1.81)	0.469	0.64 (0.23 - 1.74)	0.378

Supplemental Table 5: Uni- and multivariable Cox regression analysis results for NAT-naïve patients, including missing differentiation grade. The multivariable analysis was a complete-case analysis, except for differentiation grade. If differentiation grade was missing, this was treated as an extra category of differentiation grade. Patients who died or were censored within 90 days were excluded (N = 83). The addition of PurIST subtype to the prognostic model improved the model significantly (p = 0.0004). The effect of adjuvant treatment on overall survival was time-dependent. Tgroup 1 = 0 – 270 days, tgroup 2 = 270 days or longer. LNR = lymph node ratio. HR = hazard ratio. 95% C.I. = 95% confidence interval.

	**Univariable**		**Multivariable**	
	**HR (95% C.I.)**	**p-value**	**HR (95% C.I.)**	**p-value**
LNR = 0	Reference		Reference	
LNR > 0 & LNR < 0.2	1.19 (0.59 - 2.40)	0.632	1.73 (0.78 - 3.85)	0.180
LNR > 0.2	1.79 (0.90 - 3.57)	0.098	1.87 (0.86 - 4.06)	0.116
Well differentiated	Reference		Reference	
Differentiation grade missing	1.60 (0.38 - 6.71)	0.522	1.78 (0.37 - 8.63)	0.472
Moderately differentiated	2.85 (0.87 - 9.30)	0.082	4.66 (1.30 - 16.70)	**0.018**
Poorly differentiated	6.23 (1.81 - 21.46)	**0.004**	5.88 (1.65 – 21.0)	**0.006**
R0 resection	Reference		Reference	
R1 resection	1.32 (0.80 - 2.19)	0.279	1.24 (0.71 - 2.15)	0.455
PurIST subtype Basal-like	Reference		Reference	
PurIST subtype Classical	0.37 (0.22 - 0.64)	**< 0.001**	0.27 (0.13 - 0.55)	**< 0.001**
Adjuvant chemotherapy	Reference		Reference	
No adjuvant chemotherapy (tgroup = 1)	9.91 (3.22 - 30.50)	**< 0.001**	16.85 (5.27 - 53.90)	**< 0.001**
No adjuvant chemotherapy (tgroup = 2)	1.01 (0.43 - 2.42)	0.976	1.83 (0.71 - 4.70)	0.209

Supplemental Table 6: Uni- and multivariable Cox regression analysis results for adjuvant gemcitabine-treated patients, including missing differentiation grade. The multivariable analysis was a complete-case analysis, except for differentiation grade. If differentiation grade was missing, this was treated as an extra category of differentiation grade. Patients who died or were censored within 90 days were excluded (N = 56). The addition of PurIST subtype to the prognostic model again improved the model (p < 0.0001). LNR = lymph node ratio. HR = hazard ratio. 95% C.I. = 95% confidence interval.

	**Univariable**		**Multivariable**	
	**HR (95% C.I.)**	**p-value**	**HR (95% C.I.)**	**p-value**
LNR = 0	Reference		Reference	
LNR > 0 & LNR < 0.2	1.25 (0.59 - 2.63)	0.563	0.84 (0.37 - 1.90)	0.680
LNR > 0.2	1.59 (0.72 - 3.50)	0.248	0.74 (0.30 - 1.83)	0.518
Well differentiated	Reference		Reference	
Differentiation grade missing	1.23 (0.27 - 5.53)	0.786	4.05 (0.71 – 22.99)	0.114
Moderately differentiated	2.21 (0.66 - 7.37)	0.198	8.32 (1.77 – 39.00)	**0.007**
Poorly differentiated	5.81 (1.65 - 20.42)	**0.006**	14.2 (3.24 - 62.59)	**< 0.001**
R0 resection	Reference		Reference	
R1 resection	0.88 (0.49 - 1.57)	0.667	0.72 (0.37 - 1.41)	0.342
PurIST subtype Basal-like	Reference		Reference	
PurIST subtype Classical	0.33 (0.18 - 0.60)	**< 0.001**	0.18 (0.08 - 0.42)	**< 0.001**

Supplemental Table 7: RNA-Sequencing quality statistics.

The used reference genome was GRCh37. FASTQ files were merged per read-pair. Merged FASTQ files were cleaned by bbDUK (adapter removal and read quality clipping), as 'paired' files. Total reads reported are from before cleaning. Clean FASTQ files mapped to GRCh38 (Gencode version 48) using STAR. BAM files were assessed using *rna-seqc*. RIN values were measured using Tapestation (Agilent) in the Amsterdam UMC. Uploaded as an Excel file.

SUPPLEMENTARY FIGURES

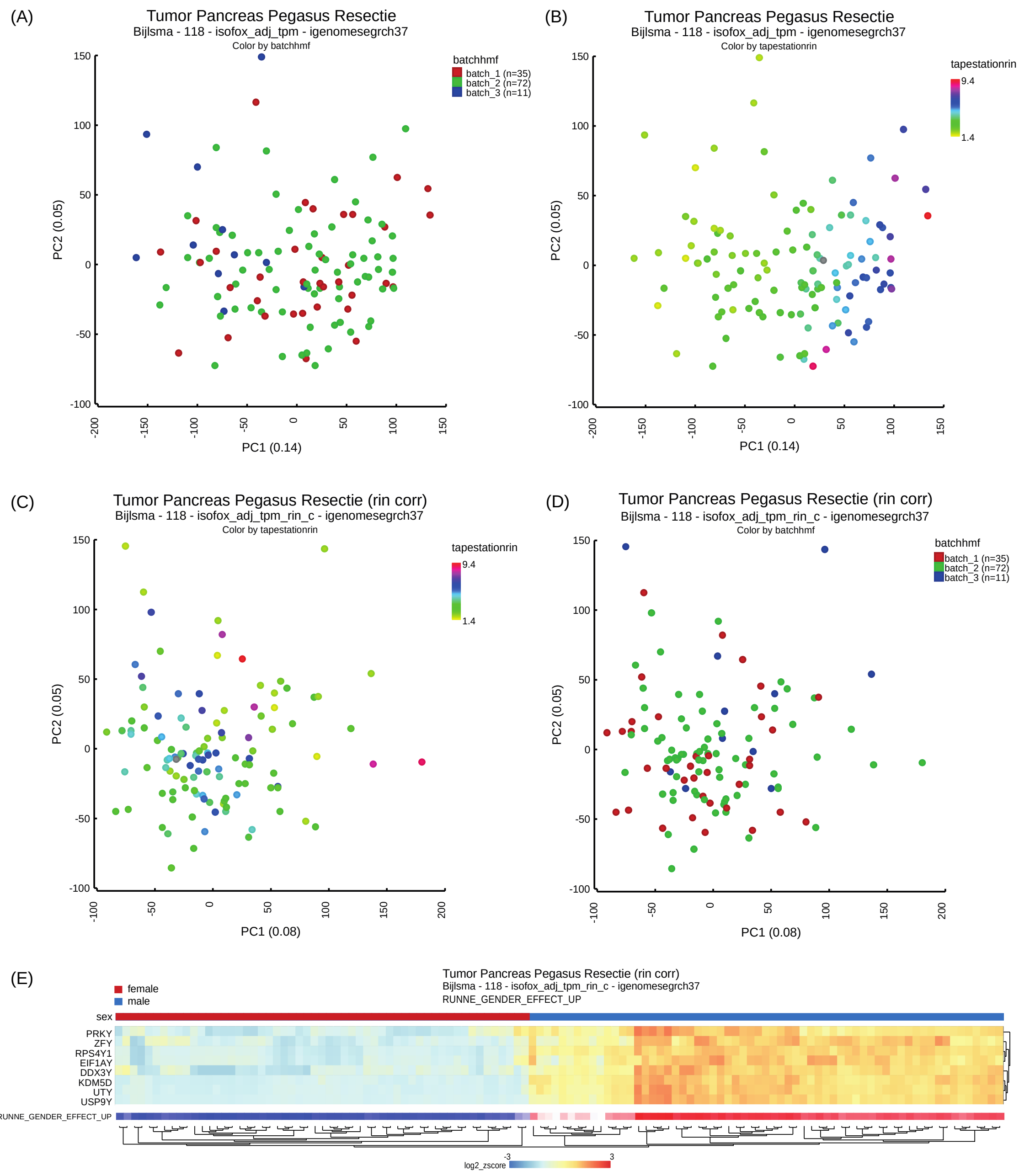

Supplemental Figure 1: Effect of RIN-value and RIN-based correction on expression levels.

A: PCA plot indicating batches in which RNA samples were submitted to HMF. Batch 3 is part of batch 2 but was sequenced separately.

B: PCA plot indicating the impact of RIN value on gene expression levels for the uncorrected RNA levels.

C: PCA plot indicating the impact of RIN value on gene expression levels for the RIN-corrected RNA levels.

D: PCA plot indicating batches in which RNA samples were submitted to HMF. Batch 3 is part of batch 2 but was sequenced separately. PCA plot was based on RIN-corrected RNA levels.

E: Z-score for a gene signature for Y-linked genes for RIN-corrected RNA levels.[21] Samples are clustered by Euclidean distance. Red = female sex, Blue = male sex.

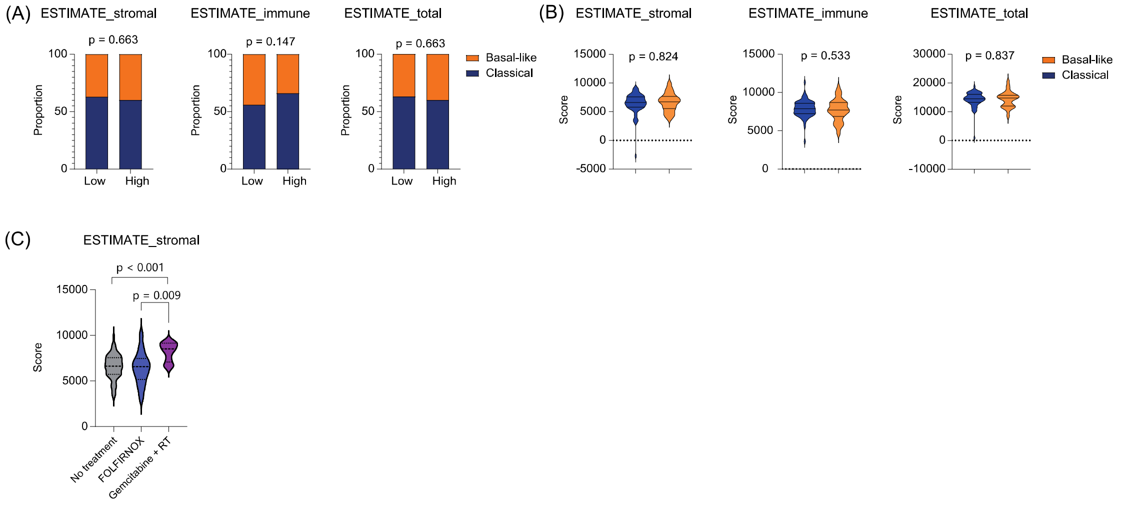

Supplemental Figure 2: ESTIMATE scores for PurIST subtypes.

Low ESTIMATE sum scores indicate a high tumor purity. Low stromal or immune scores indicate a small stromal or immune component.

A: Subtype proportion in samples with low and high ESTIMATE scores. Samples were categorized based on median ESTIMATE scores. P-values by Chi square test.

B: ESTIMATE scores in classical and basal-like subtype tumors. P-values by Mann-Whitney U test.

C: ESTIMATE stromal scores for neoadjuvant treatment regimens. P-values by Mann-Whitney U test.

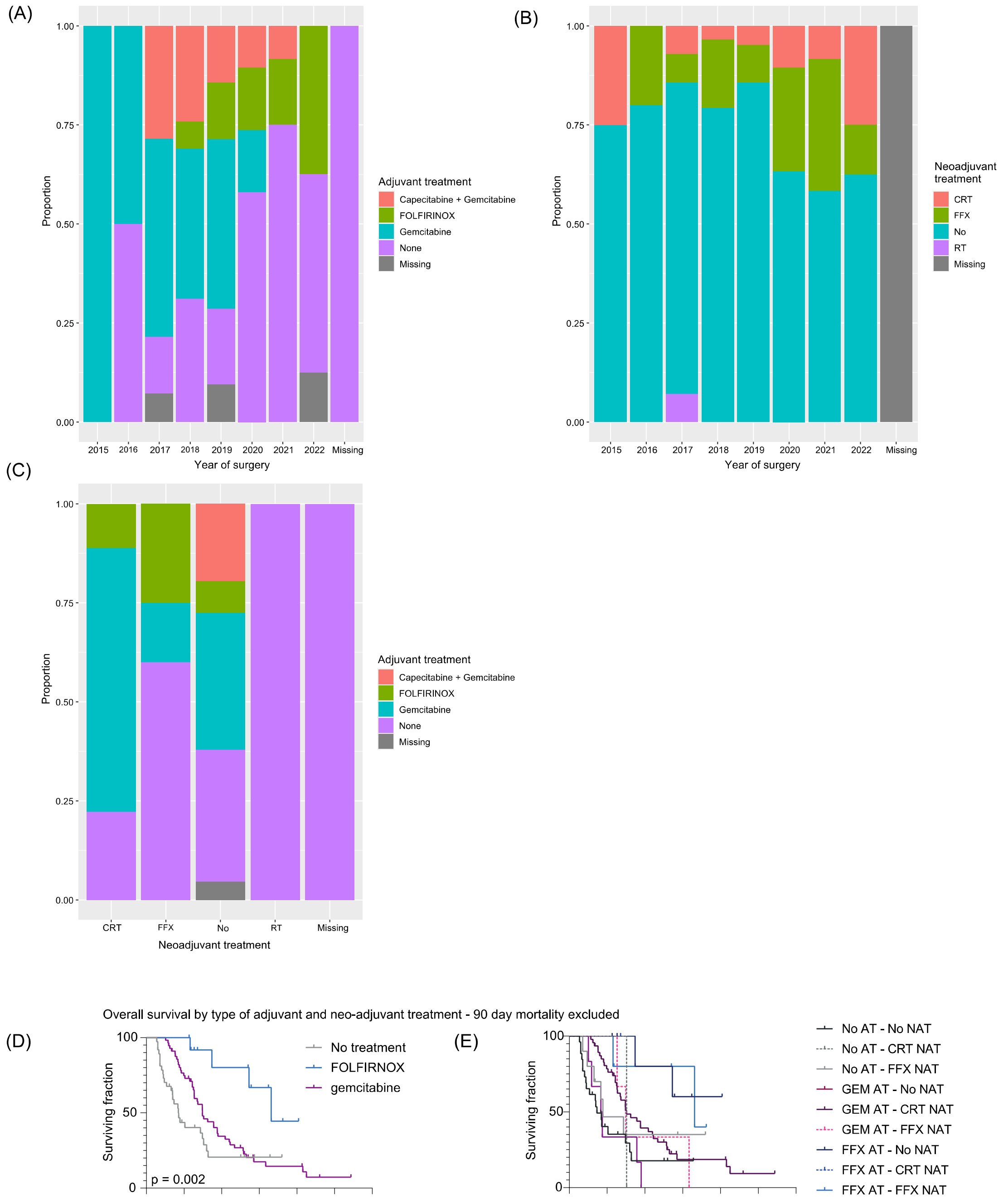

Months	0	12	24	36	48	60	72
NT	37	13	5	2	0	0	0
censored	0	4	6	9	11	11	11
FFX	13	13	7	4	1	0	0
censored	0	0	4	6	8	9	9
GEM	56	41	18	6	5	1	0
censored	0	1	3	7	7	9	10

Months	0	12	24	36	48	60	72
no AT - no NAT	26	8	3	1	0	0	0
no AT - CRT	1	1	0	0	0	0	0
no AT - FFX	10	4	2	1	0	0	0
GEM AT - no NAT	47	36	17	5	5	1	0
GEM AT - CRT NAT	6	2	0	0	0	0	0
GEM AT - FFX NAT	3	3	1	1	0	0	0
FFX AT - no NAT	7	7	4	2	1	0	0
FFX AT - CRT NAT	1	1	0	0	0	0	0
FFX AT - FFX NAT	5	5	3	2	0	0	0

Supplemental Figure 3: Neoadjuvant and adjuvant treatment regimens in the RNA cohort.

A: Distribution of adjuvant treatment regimens per year of surgery.

B: Distribution of neoadjuvant treatment regimens per year of surgery.

C: Distribution of adjuvant treatment regimens per neoadjuvant treatment regimen. CRT = chemotherapy (gemcitabine) + radiotherapy, FFX = FOLFIRINOX, RT = radiotherapy.

D: Overall survival after surgery for adjuvant treatment types. Patients who died or were censored within 90 days were excluded. Overall the Log-Rank test is indicated.
E: Overall survival after surgery for adjuvant and neoadjuvant treatment types. Patients who died or were censored within 90 days were excluded. Overall the Log-Rank test is indicated. AT = adjuvant therapy, NAT = neoadjuvant therapy, CRT = chemotherapy (gemcitabine) + radiotherapy, GEM = gemcitabine or gemcitabine + capecitabine, FFX = FOLFIRINOX.

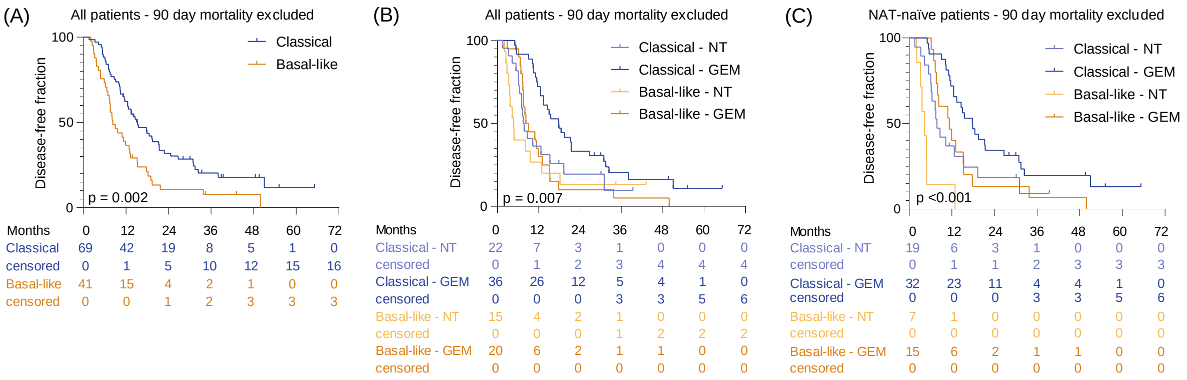

Supplemental Figure 4: Disease-free survival after surgery for transcriptome-based subtypes.

Patients who died or were censored within 90 days were excluded. Overall the Log-Rank test is indicated. Numbers of patients at risk and censored patients are provided.

A: DFS for the complete cohort (n = 110) per RNA subtype.

B: DFS for patients treated with adjuvant gemcitabine (with or without capecitabine, n = 56) compared to patients without adjuvant treatment (n = 37).

C: DFS for patients who did not receive NAT treated with adjuvant gemcitabine (with or without capecitabine, n = 47) compared to patients without adjuvant treatment (n = 26).
